# Higher Vitamin D Levels before Methotrexate Therapy Initiation Are Associated with Lower Subsequent Mortality in Patients with Rheumatoid Arthritis

**DOI:** 10.3390/nu16030401

**Published:** 2024-01-30

**Authors:** Shahdi K. Malakooti, Hinnah Siddiqui, Brigid Wilson, Taissa Bej, Megan O’Mara, Alexandra Desotelle, Alyssa Lange, Carey L. Shive, Nora G. Singer, Grace A. McComsey, Lenche Kostadinova, Maya Mattar, David A. Zidar, Donald D. Anthony

**Affiliations:** 1 Case Western Reserve University School of Medicine, Cleveland, OH 44106, USA; 2 Department of Medicine, MetroHealth Medical Center, Cleveland, OH 44109, USA; 3 Louis Stokes Cleveland Veterans Affairs Medical Center, Cleveland, OH 44106, USA; hinnah.siddiqui@va.gov (H.S.); brigid.wilson@va.gov (B.W.);; 4 University Hospitals Cleveland Medical Center, Cleveland, OH 44106, USA

**Keywords:** autoimmune disease, rheumatoid arthritis, mortality, survival, vitamin D

## Abstract

(1) Vitamin D deficiency is associated with mortality in the general population and has been observed in one rheumatoid arthritis (RA) cohort. Here, we investigate the relationship between 25-hydroxyvitamin D (25(OH)D) levels before methotrexate (MTX) therapy initiation in patients with RA and the subsequent all-cause mortality in a national Veterans Affairs (VA) cohort. (2) This is a retrospective study on RA patients time-oriented around the initial MTX prescription and 25(OH)D levels before starting MTX. We examined survival in patients with 25(OH)D levels > 50 nmol/L and ≤50 nmol/L using the Cox Proportional Hazard Model and fully adjusted for risk factors. (3) In total, 15,109 RA patients were included in the nationwide cohort. RA patients with 25(OH)D levels > 50 nmol/L before starting MTX had a 28% reduced risk of mortality when compared to those with levels ≤ 50 nmol/L (HR: 0.72, CI: 0.64–0.80, *p* < 0.001) after adjusting for traditional risk factors. (4) In this national RA cohort receiving standard-of-care MTX, patients with 25(OH)D levels > 50 nmol/L have a lower subsequent mortality when compared to those with 25(OH)D levels ≤ 50 nmol/L. It remains to be determined whether increasing Vitamin D levels in RA patients initially found to be Vitamin D deficient impacts their all-cause mortality.

## 1. Introduction

Vitamin D is a hormone with immune-modulating effects [1]. While Vitamin D is traditionally known for its impact in bone homeostasis, it also regulates immune function in multiple domains including autoimmune disease such as rheumatoid arthritis (RA) [2]. From a patient perspective, there is increasing interest in “natural” or adjunct treatment options for autoimmune disease including RA. However, clinicians face a paucity of clinical guidelines regarding target Vitamin D levels in immune health. Vitamin D is associated with the Disease Activity Score (DAS28) and Health Assessment Questionnaire Disability Index (HAQ-DI) in patients with early RA [3,4], and with serum C-reactive protein (CRP) levels in RA and the general population [5,6]. In a meta-analysis of randomized placebo-controlled trials of Vitamin D supplementation in RA patients, higher doses (>50,000 IU) of Vitamin D supplementation improved the DAS28 levels [7]. Further, Vitamin D supplementation reduced incident autoimmune disease in a randomized placebo-controlled trial when compared to a placebo [8].

Mortality is associated with Vitamin D levels in several groups. In the general population, lower 25-hydroxyvitamin D (25(OH)D) levels correlate with increased subsequent mortality [9,10]. In patients with hyperlipidemia, lower serum 25(OH)D levels are independently associated with all-cause and cardiovascular mortality [11]. In cohorts of patients with diabetes [12,13] and known cardiovascular disease [14], higher levels of 25(OH)D are associated with a lower risk of mortality. Further, in nonalcoholic fatty liver disease, lower Vitamin D levels are again associated with increased mortality [15]. Finally, in one study of individuals with RA, serum 25(OH)D levels < 37.30 nmol/L are associated with an increased risk of all-cause mortality [16].

In a national Veterans Affairs (VA) study of patients with RA, we investigated the relationship between 25(OH)D levels before methotrexate (MTX) therapy initiation, the first line of disease modifying therapy for RA, and subsequent mortality. We adjusted for traditional cardiovascular risk factors, including age, sex, race and ethnicity, smoking status, body mass index (BMI), statin use, and the Charlson comorbidity index (CCI). For the local VA cohort, we conducted chart adjudication for Vitamin D supplementation and evaluated the prevalence of Vitamin D supplementation in this cohort.

## 2. Materials and Methods

### 2.1. Participants and Study Design

This is a retrospective study of national VA patients with an ICD-9/10 diagnosis of RA observed during Rheumatology clinic visits. It was approved by the Institutional Review Board of the Cleveland Louis Stokes VA Medical Center, Protocols 17046-H35 and CY19-004. We restricted the study population to adult RA patients with an ICD-9 or ICD-10 diagnosis of RA made during Rheumatology clinic visits, initial MTX prescription filled between 1 January 2006 and 31 December 2019, at least a 90-day supply of MTX filled, a MTX medication possession ratio of at least 75%, and a 25(OH)D level obtained within one year prior to starting MTX (*N* = 15,109). Patients were followed until death, their most recent encounter with the VA, or until 2020 (whichever came first). We examined survival in groups of RA patients with 25(OH)D levels available in the year prior to MTX initiation, stratifying these patients by serum levels of 25(OH)D >50 nmol/L (>20 ng/mL) and ≤50 nmol/L (≤20 ng/mL).

Additionally, we performed a chart review of a local RA cohort at the Cleveland VA including the very first Vitamin D level in the VA system (*n* = 197) and Vitamin D level within one year before starting MTX (*n* = 105), in which we verified medications, diagnoses, and Vitamin D supplementation at multiple time points.

### 2.2. Data Extraction and Statistical Analysis

Data were extracted from the VA Corporate Data Warehouse (CDW) and accessed using the VA Informatics and Computing Infrastructure (VINCI) as described in Lange et al. [17]. For both the nationwide and local populations, we obtained the available clinical immunoassay serum 25(OH)D levels. Vitamin D level assessment included clinical immunoassay 25(OH)D levels before (earliest and within one year prior to MTX initiation) and after MTX therapy (three to 12 months after MTX initiation). For the nationwide cohort, we extracted demographics, comorbidities, and additional cardiovascular risk factors at time of MTX initiation. The primary outcome was mortality obtained from VA medical records supplemented by vital status information from the Social Security Administration and Centers for Medicare & Medicaid Services.

Data queries and statistical analyses were completed with SQL Server Management Studio (SSMS) 18.0 and R software v 4.0.5 (R Core Team, Vienna, Austria). No data imputation was performed. All *p*-values presented are two-sided and unadjusted. Information not available is labeled as unknown. We examined all-cause mortality in patients with 25(OH)D > 50 nmol/L and ≤50 nmol/L using the Cox Proportional Hazard Model. Patients with 25(OH)D levels ≤ 50 nmol/L were selected as the reference group. The time to event was calculated from the date of MTX initiation to the death date, with follow-up ending on 31 December 2019. There were no missing covariate values. The model was fully adjusted for traditional cardiovascular risk factors, including age, sex, race and ethnicity, smoking status, body mass index (BMI), statin use, and the Charlson comorbidity index (CCI). BMI was categorized according to the CDC guidelines: underweight, <18.5; healthy weight, 18.5 to 25; overweight, 25.0 to 30; obese, 30.0 or higher. We also compared paired binary outcomes with the McNemar Test by treatment periods in paired observations between the proportion of 25(OH)D levels > 50 nmol/L at baseline, pre-MTX, and post-MTX in the national cohort. Within the local Cleveland cohort, we evaluated Vitamin D supplementation and the proportion of patients with 25(OH)D levels > 50 nmol/L.

## 3. Results

In total, 15,109 patients fulfilled diagnostic criteria for RA with an ICD-9 or ICD-10 diagnosis of RA made at a VA Rheumatology clinic visit with a 25(OH)D level within one year before MTX initiation, an initial MTX prescription filled between 1 January 2006 and 31 December 2019, at least a 90-day supply of MTX filled, and a MTX medication possession ratio of at least 75% in the national cohort. National patient characteristics at MTX initiation according to groups of serum 25(OH)D levels are shown in Table 1. The patient median age was 63 (IQR 55, 69) and included 12,344 males (82%) and 2765 females (18%). In the year before MTX initiation, 25(OH)D levels ≤ 50 nmol/L were present in 2823 (19%) and levels > 50 nmol/L were present in 12,286 (81%) of patients. Compared to patients with 25(OH)D serum concentrations ≤ 50 nmol/L, individuals with 25(OH)D levels > 50 nmol/L were older, less frequently female, less frequently Black, less frequently Hispanic or Latino, less frequently obese, less frequently smokers, had higher and broader ranges of Charlson scores, and more frequently on a statin. In the local Cleveland VA cohort, clinical characteristics were similar to those of the national cohort (Table 2).

### 3.1. Subsequent Mortality Risk Is Lower in RA Patients with Higher 25(OH)D Levels before MTX Initiation

**Figure 1:**
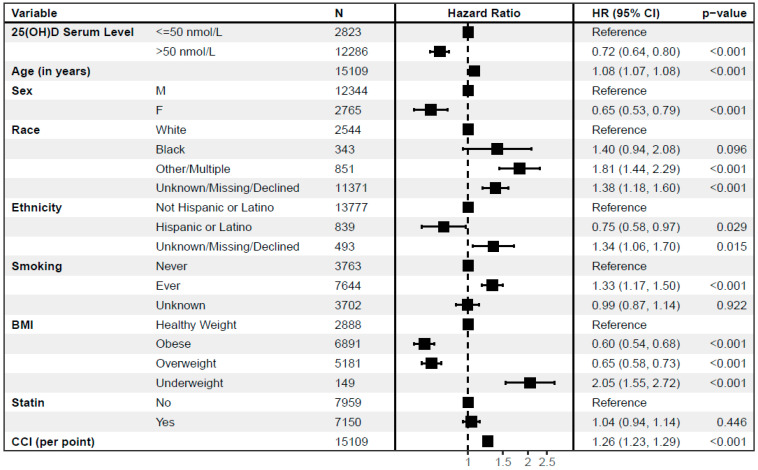
Patients with higher 25(OH)D levels before methotrexate initiation have a lower subsequent mortality risk in the adjusted national survival model.

Figure 1 shows adjusted hazard ratios for all-cause mortality for individuals by serum 25(OH)D levels > 50 nmol/L compared to ≤50 nmol/L (reference) in the national cohort. Individuals with 25(OH)D levels > 50 nmol/L exhibited a reduced hazard for subsequent all-cause mortality (HR 0.72, CI: 0.64–0.80, *p* < 0.001) in the fully adjusted model. Every additional year of age (1.08 per year, 1.07–1.08, *p* < 0.001), if ever a smoker (1.33, 1.17–1.50, *p* < 0.001), a greater Charlson comorbidity index (1.26 per unit increase, 1.23–1.29, *p* < 0.001), and an underweight BMI (2.05, 1.55–2.72, *p* < 0.001) correlated with an increased mortality hazard. Female sex (HR 0.65; 0.53–0.79; *p* < 0.001) and Hispanic or Latino ethnicity (0.75, 0.58–0.97, *p* = 0.029) exhibited a reduced hazard for all-cause mortality. Overweight BMI was associated with reduced hazard for mortality (0.65, 0.58–0.73, *p* < 0.001), and obese BMI was associated with further reduction in mortality risk (0.60, 0.54–0.68, *p* < 0.001).

### 3.2. Proportion of Patients with 25(OH)D Levels > 50 nmol/L Increases before Methotrexate Initiation

**Figure 2:**
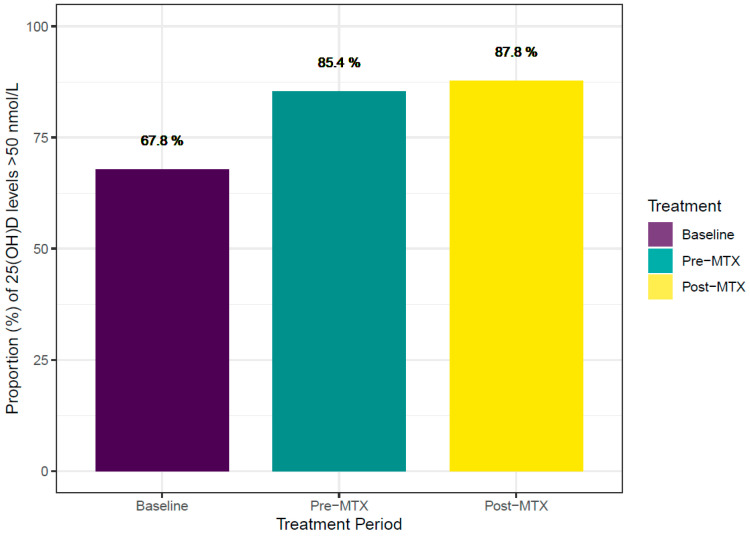
Proportions of patients with 25(OH)D levels > 50 nmol/L increase across time points in the national cohort. Proportions of patients with 25(OH)D levels across time points with three clinical measurements of 25(OH)D at baseline (first available measurement), pre-MTX (in the year prior to methotrexate initiation), and post-MTX (3 to 12 months after methotrexate initiation); *n =* 2873 patients. Y axis; the proportion of patients with 25(OH)D levels > 50 nmol/L.

To understand whether Vitamin D levels change either before or after MTX initiation, we next looked at 25(OH)D levels longitudinally in a subgroup where these levels were available. In the national VA cohort, the proportions of patients with 25(OH)D levels over 50 nmol/L at baseline (first available measurement), pre-MTX (in the year prior to MTX initiation), and post-MTX (3 to 12 months after MTX initiation) were 67.8%, 85.4%, and 87.8%, respectively (Figure 2). We also compared paired binary outcomes with the McNemar Test by treatment periods in paired observations between the proportion of 25(OH)D levels > 50 nmol/L and 25(OH)D levels at baseline, pre-MTX, and post-MTX. The proportion of patients with 25(OH)D levels > 50 nmol/L differed significantly between baseline and pre-MTX (*p* < 0.001) and between pre-MTX and post-MTX (*p =* 0.001) in the national cohort.

In the local Cleveland VA cohort, the proportions of patients with clinical measurements of 25(OH)D levels > 50 nmol/L at baseline and pre-MTX (*n* = 48) were somewhat similar to those in the national cohort (76.8% and 82.1%). Vitamin D supplementation data were obtained through a chart review; 41% were receiving Vitamin D supplementation at baseline, while 85% were receiving Vitamin D supplementation (bolus or daily supplementation) 3 to 12 months after MTX initiation (post-MTX).

## 4. Discussion

Here, we observe that higher Vitamin D levels, in the year before standard-of-care MTX initiation, correlate with lower subsequent mortality in the national VA RA population. This relationship holds after adjusting for traditional cardiovascular risk factors. A number of prior studies have linked Vitamin D levels to mortality in the general population [9,10] and in one other study in terms of RA [16]. Here, we extend these findings, for the first time, in a large and well-characterized national VA RA population from the reference point of standard-of-care MTX therapy initiation.

Vitamin D levels may link to mortality through cardiovascular or immune modulating mechanisms. In non-RA cohorts, lower Vitamin D levels were associated with increased cardiovascular disease (CVD) events in multiple studies [18,19,20]. It is therefore thought that the relation between lower Vitamin D levels and mortality in the general population is in large part attributable to cardiovascular events. Certainly, the relation observed here may be in part mediated by linkage between Vitamin D and cardiovascular events. In this regard, risk of cardiovascular events is increased in RA, and this risk is thought to be driven by multiple factors, including pathogenic inflammation [21]. The specific effects of Vitamin D on inflammation include the following: (A) In RA synovial fluid, Vitamin D attenuates NOD-, LRR-, and pyrin domain-containing protein 3 (NLRP3), Toll-like receptor 1 (TLR1), and TLR4 expression [22]; (B) Vitamin D encourages the transition of macrophages from proinflammatory M1 to the M2 state through the STAT-1/TREM-1 pathway [23,24,25]; and (C) Vitamin D supplementation ameliorates DAS28 and ESR levels, and thus, an altered Vitamin D metabolism may also contribute to rheumatoid disease severity [7]. In regard to the latter, synovial fluid analyses from individuals with RA compared with controls reveal a reduction in Vitamin D metabolite 3-epi-25(OH)D3 in RA, but not in other Vitamin D metabolites [26].

Regardless of the specific mechanism linking Vitamin D and mortality, Vitamin D supplementation has been shown to reduce mortality in multiple cohorts. Further, heterogeneity in Vitamin D supplementation strategies (daily vs. monthly) may have differential effects on subsequent survival. The VITALS trial, a randomized placebo-controlled clinical trial including daily dosing of Vitamin D, demonstrates a decreased incidence of autoimmune disease in the arm receiving Vitamin D supplementation, and this arm also shows a reduction in total cancer mortality [27]. The D-health clinical trial, a randomized placebo-controlled clinical trial of bolus monthly Vitamin D supplementation, does not show reduction in all-cause mortality in the Vitamin D-supplemented group [28], which may be due to bolus and infrequent scheduling of Vitamin D supplementation. Indeed, a recent meta-analysis of Vitamin D supplementation strategies on clinical trials demonstrates reduced mortality risk with daily dosing compared to bolus dosing [29]. Thus, supplementation strategies (daily vs. intermittent) may be of high importance. Lastly, in a large national VA study, the supplementation of Vitamin D before COVID-19 infection correlates with lower mortality [30].

Other factors associated with mortality in this national RA cohort include age, smoking status, sex, the Charlson comorbidity index, BMI, and ethnicity. In our study, underweight individuals had increased risk of subsequent all-cause mortality, which may be related to weight loss. The mortality risk was reduced in the higher BMI groups, and most pronounced in RA patients with obesity. This has been addressed within the VA RA cohort previously, where after considering peak life BMI, current weight, and rate of annual weight loss in patients with RA, it was observed that a lower current weight and rate of weight loss were associated with greater mortality, and the latter was in a dose-dependent fashion where greater weight loss/year was associated with greater mortality, even after adjustment for RA disease activity parameters CRP and HAQ. At the same time, peak BMI was not associated with mortality [31]. This observed relation between greater weight loss and greater mortality is thought to at least partially explain the inverse association between BMI and mortality in RA. Whether obesity may lend an additional dose-dependent protective effect in disease activity (independent of CRP or HAQ) or reflect social drivers of health is yet to be determined.

Further, whether the effect of Vitamin D supplement is mediated through the lowering of cardiovascular risk or driven by multiple pathways is unknown. In our national cohort, when looking at Vitamin D levels over time, we observed that the proportions of individuals with Vitamin D levels above 50 nmol/L increased before initiating MTX. This may have been due to Vitamin D supplementation. While we are unable to adequately assess the Vitamin D supplement status in our national RA cohort, within our local cohort, we have carefully looked at the Vitamin D supplement status, and we observed 41% of people on supplements at the first Vitamin D level before MTX initiation, while after MTX initiation, 85% were on Vitamin D supplements. Nevertheless, these data are at least consistent with Vitamin D supplementation being common and feasible in this patient population. While many societies have endorsed levels for Vitamin D sufficiency based on studies on bone homeostasis, in which lower Vitamin D levels are associated with fractures and reduced bone health, the optimal Vitamin D levels for immune health in RA are yet to be determined. These issues will need to be considered in future supplementation trials.

Limitations to our study include the observational retrospective nature of both the nationwide and local RA cohorts. Traditionally, 25(OH)D levels are checked for bone health per clinical guidelines. It is likely that people with low Vitamin D levels at the first check are overrepresented in patients with serial 25(OH)D testing in the longitudinal analysis. However, the mortality findings here did not require longitudinal Vitamin D levels and are not affected by this limitation. Further, in the VA system, the reporting of 25(OH)D clinical lab results vary, with some locations previously providing precise numeric values only when >20 ng/mL (50 nmol/L), and some, but not all, reporting numeric values when ≤20 ng/mL (50 nmol/L) as well. Thus, we were able to analyze 25(OH)D values only as above or below this threshold (20 ng/mL) in our nationwide cohort without the precision to address incremental changes. However, this threshold is in alignment with 25(OH)D levels ≤ 50 nmol/L that are defined as insufficient or deficient for Vitamin D by most societal guidelines for bone health. We were also not able to evaluate supplementation in relation to paired Vitamin D levels in the local chart-adjudicated cohort. Lastly, we were not able to evaluate for social drivers of health that may influence Vitamin D levels and survival outcomes in these groups. In spite of these limitations, our overall observation of lower Vitamin D levels at the time of first-line therapy (MTX) initiation for RA associating with greater mortality indicates another link between Vitamin D homeostasis and bio-immune health. We have also recently defined the absolute lymphocyte count and red cell distribution width to associate with mortality in this cohort [17].

An additional strength of this study is the additional assurance of RA diagnoses by focusing on patients with RA diagnoses made during Rheumatology clinic visits as part of the inclusion criteria, and locally verified via chart adjudication. Finally, orientation before standard-of-care MTX initiation, the first line of treatment for RA, provides a common time point that avoids potential interactions between MTX and Vitamin D, and an indication that RA was active and necessitated treatment.

## 5. Conclusions

Higher Vitamin D levels prior to the initiation of MTX are associated with lower subsequent mortality in patients with RA. Mechanisms underlying this relationship are yet to be determined, and future trials on Vitamin D supplementation are needed to understand how Vitamin D status affects immune health and mortality risk in autoimmune diseases.

## Figures and Tables

**Table 1:** Patient characteristics stratified by 25(OH)D level before methotrexate initiation in the national VA cohort.

	Overall *N* = 15,109	25(OH)D ≤ 50 nmol/L *n* = 2823	25(OH)D > 50 nmol/L *n* = 12,286	*p*-Value
Age (years) at Index (IQR)	63 (55, 69)	59 (51, 66)	64 (56, 70)	<0.001
Sex, *n* (%)				<0.001
Female	2765 (18%)	638 (23%)	2127 (17%)	
Male	12,344 (82%)	2185 (77%)	10,159 (83%)	
Race, *n* (%)				<0.001
Black	2544 (17%)	839 (30%)	1705 (14%)	
Other/Multiple	343 (2.3%)	81 (2.9%)	262 (2.1%)	
Unknown/Missing/Declined	851 (5.6%)	181 (6.4%)	670 (5.5%)	
White	11,371 (75%)	1722 (61%)	9649 (79%)	
Ethnicity, *n* (%)				0.034
Hispanic or Latino	839 (5.6%)	185 (6.6%)	654 (5.3%)	
Not Hispanic or Latino	13,777 (91%)	2550 (90%)	11,227 (91%)	
Unknown/Missing/Declined	493 (3.3%)	88 (3.1%)	405 (3.3%)	
BMI, *n* (%)				<0.001
<18.5	149 (1.0%)	36 (1.3%)	113 (0.9%)	
18.5 to <25	2888 (19%)	511 (18%)	2377 (19%)	
25 to <30	5181 (34%)	822 (29%)	4359 (35%)	
≥30	6,891 (46%)	1454 (52%)	5437 (44%)	
Smoking, *n* (%)				0.001
Ever a Smoker	7644 (51%)	1510 (53%)	6134 (50%)	
Never a Smoker	3763 (25%)	685 (24%)	3078 (25%)	
Unknown	3702 (25%)	628 (22%)	3074 (25%)	
Charlson Comorbidity Index, Median (IQR)	1.00 (1.00, 3.00)	1.00 (1.00, 2.00)	1.00 (1.00, 3.00)	<0.001
Statin, *n* (%)				<0.001
No Statin	7959 (53%)	1706 (60%)	6253 (51%)	
Statin Use	7150 (47%)	1117 (40%)	6033 (49%)	

BMI, body mass index; IQR, interquartile range; Data expressed as median (IQR) or number (%); BMI < 18.5 = Underweight, 18.5 to <25 = Healthy Weight, 25.0 to <30 = Overweight, ≥30 = Obese.

**Table 2:** Patient characteristics stratified by 25(OH)D level before methotrexate initiation in the local VA cohort.

	Overall * N = 105	25(OH)D ≤ 50 nmol/L *n* = 18	25(OH)D > 50 nmol/L *n* = 87	*p*-Value
Age (years) at Index (IQR)	66 (60, 70)	63 (51, 67)	67 (62, 70)	0.03
Sex, *n* (%)				0.07
Female	10 (9.5%)	4 (22%)	6 (6.9%)	
Male	95 (90%)	14 (78%)	81 (93%)	
Race, *n* (%)				0.4
Black	7 (6.7%)	2 (11%)	5 (5.7%)	
Other/Multiple	3 (2.9%)	1 (5.6%)	2 (2.3%)	
Unknown/Missing/Declined	5 (4.8%)	1 (5.6%)	4 (4.6%)	
White	90 (86%)	14 (78%)	76 (87%)	
Ethnicity, *n* (%)				0.07
Hispanic or Latino	1 (1.0%)	1 (5.6%)	0	
Not Hispanic or Latino	102 (97%)	16 (89%)	86 (99%)	
Unknown/Missing/Declined	2 (1.9%)	1 (5.6%)	1 (1.1%)	
BMI, *n* (%)				0.3
≤18.5	0			
18.5 to <25	14 (13%)	2 (11%)	12 (14%)	
25 to <30	36 (34%)	4 (22%)	32 (37%)	
≥30	53 (50%)	11 (61%)	42 (48%)	
Unknown	2 (1.9%)	1 (5.6%)	1 (1.1%)	
Smoking, *n* (%)				0.2
Ever a Smoker	84 (80%)	13 (72%)	71 (82%)	
Never a Smoker	19 (18%)	4 (22%)	15 (17%)	
Unknown	2 (1.9%)	1 (5.6%)	1 (1.1%)	
Statin, *n* (%)				0.01
No Statin	43 (41%)	12 (67%)	31 (36%)	
Statin Use	62 (59%)	6 (33%)	56 (64%)	

* 92 patients initiated on methotrexate did not have a 25(OH)D level checked in the year before methotrexate initiation. BMI, body mass index; IQR, interquartile range; Data expressed as median (IQR) or number (%); BMI < 18.5 = Underweight, 18.5 to <25 = Healthy Weight, 25.0 to <30 = Overweight, >30 = Obese.

## Data Availability

The original contributions presented in the study are included in the article material. The data are publicly available upon request to the corresponding authors.
